# Improving the quality of trials reporting

**DOI:** 10.1002/nop2.1089

**Published:** 2021-11-23

**Authors:** Debra Jackson, Mark Hayter, Diana Baptiste

**Affiliations:** ^1^ The University of Sydney Sydney NSW Australia; ^2^ Manchester Metropolitan University Manchester UK; ^3^ Johns Hopkins University School of Nursing Baltimore MD USA

Over the last few years, there has been an increase in studies that use a clinical trial‐type design to measure the effects of a nursing a intervention. This marks a positive step in nursing science and the evidence base for the efficacy of nursing. However, whilst we welcome this, it does highlight that nurse researchers can often be relatively new to the conduct of clinical trials. This creates an issue where the importance of encouraging this type of work must also be balanced with ensuring the studies are conducted rigorously and meet with the international standards expected of this type of research.

The AllTrials initiative (alltrials.net) calls for the prospective registration of all clinical trials on a trials registry and for full reporting of the methods and findings of trials. This is important because failing to fully report results can have a range of serious consequences including distortion and misrepresentation of results as well as ethical implications associated with non‐transparent reporting. This important initiative has provided a catalyst for on‐going review and re‐examination of how trials are assessed for publication. Some advancements are evident, particularly in raised awareness of the purpose of trial registries and the need for prospective trial registration. While this is an issue that seems to be improving—lack of registration—prospective or not at all—is still a common reason for rejection.

There are additional areas of concern with papers (reporting trials) that are not fully adherent to the requirements of the AllTrials initiative still making it through to publication (Gray et al., 2017; Gray, Brown & Gray, 2019; Gray, Gray & Brown, 2019; Gray & Mackay, 2020; Noyes, 2018, 2021). Noyes (2021) has recently reported an analysis of the most common issues in trials published by the *Journal of Advanced Nursing*. These included registration issues, renaming of trials in ways to avoid the responsibilities of registration and transparent reporting, inconsistencies between the trial as registered and the trial as reported, selective reporting, altering primary and secondary outcomes, sample size differences, reporting the outcomes of a single trial over multiple papers and inconsistencies not being picked up in the peer review process. This final issue is previously noted in the literature (Chauvin et.al., 2015). Failure to publish is another issue of concern, with one recent study finding that only 41% of clinical trials were reported within the recommended time frame and 36% were not reported at all (DeVito et.al, 2020).

As journal editors‐in‐chief (*Journal of Advanced Nursing*/*JAN*, Debra Jackson; *Journal of Clinical Nursing*/*JCN*, Mark Hayter; *Nursing Open*/*NOP,* Diana Baptiste), we know that we have a crucial role to play in contributing to improvements in trial reporting. At *JAN, JCN* and *NOP,* we have worked together to make some changes that we think will help us to make real strides in this important area. We have initiated new processes for picking up inconsistencies in relation to trial registration and reporting, extended the word limit of papers to remove any need for reporting of trial outcomes across multiple papers and produced additional guidance for peer reviewers reviewing papers reporting trials. One substantial change we have made is ensuring reviewers get access to the original trial registration document associated with the paper. This means the manuscript will be unblinded, but this is made clear to authors and this improvement will add to the rigour of the review process. Chauvin et al. (2015) have previously drawn attention to the importance of clear guidelines for peer reviewers of papers reporting trials. In this new process, we are being intentionally transparent to improve adherence to guidelines at each stage of the process. We have outlined the updated processes in revised author guidelines for all three journals. Additionally, we are planning to do regular analysis of trial reporting which we will make available to readers, authors and reviewers.

The aforementioned endeavours support our dedication to consistently publishing papers that report ethical and sound research that honours integrity and full transparency. As journal editors‐in‐chief of *JAN, JCN* and *NOP,* we have the privilege of working together to promote prospective registration, increase opportunity for timely reporting of outcomes and improve the overall quality of trials reporting in the three publications. We are hopeful the research and work we are doing will help improve publication of trials for the global nursing community and welcome further feedback on ways we can better adhere to trial reporting requirements and support triallists in bringing their work through to publication.

## References

[nop21089-bib-0001] Chauvin, A. , Ravaud, P. , Baron, G. , Barnes, C. , & Boutron, I. (2015). The most important tasks for peer reviewers evaluating a randomized controlled trial are not congruent with the tasks most often requested by journal editors. BMC Medicine, 13, 158. 10.1186/s12916-015-0395-3 26141137 PMC4491236

[nop21089-bib-0002] DeVito, N. J. , Bacon, S. , & Goldacre, B. (2020). Compliance with legal requirement to report clinical trial results on ClinicalTrials.gov: A cohort study. The Lancet, 395(10221), 361–369. 10.1016/S0140-6736(19)33220-9 31958402

[nop21089-bib-0003] Gray, R. , Badnapurkar, A. , & Thomas, D. (2017). Reporting of clinical trials in nursing journals: How are we doing? Journal of Advanced Nursing, 73, 2782–2784. 10.1111/jan.13149 27627197

[nop21089-bib-0004] Gray, R. , Brown, E. , & Gray, G. (2019). A review of prospective trial registration in the *Journal of Advanced Nursing* in 2018. Journal of Advanced Nursing, 75, 2051–2053. 10.1111/jan.14090 31162699

[nop21089-bib-0005] Gray, R. , Gray, G. , & Brown, E. (2019). A review of prospective registration of trials published in nursing science journals in 2017. Journal of Advanced Nursing, 75, 3263–3271. 10.1111/jan.14131 31237000

[nop21089-bib-0006] Gray, R. , & Mackay, B. (2020). A review of prospective registration of randomized controlled trials published in the *Journal of Advanced Nursing* in 2019. Journal of Advanced Nursing, 76, 1491–1493. 10.1111/jan.14345 32144820

[nop21089-bib-0007] Noyes, J. (2018). Which studies should be registered on a clinical trials registry? Journal of Advanced Nursing, 74, 2479. 10.1111/jan.13696 29732619

[nop21089-bib-0008] Noyes, J. (2021). The trials and tribulations of trial registration and reporting: Why are some nursing trials still slipping through the net? Journal of Advanced Nursing. 10.1111/jan.15038 34636063

